# Apolipoprotein E and Alzheimer’s Disease in Italian Population: Systematic Review and Meta-Analysis

**DOI:** 10.3390/brainsci14090908

**Published:** 2024-09-07

**Authors:** Diana Marisol Abrego-Guandique, Giorgia Francesca Saraceno, Roberto Cannataro, Marilyn Manzzo de Burnside, Maria Cristina Caroleo, Erika Cione

**Affiliations:** 1 Department of Health Sciences, University of Magna Graecia Catanzaro, 88100 Catanzaro, Italy; 2 Department of Pharmacy, Health and Nutrition Sciences, University of Calabria, 87036 Rende, Italy; giorgiafrancesca.saraceno@unical.it (G.F.S.); erika.cione@unical.it (E.C.); 3 Galascreen Laboratories, University of Calabria, 87036 Rende, Italy; r.cannataro@gmail.com; 4 Research Division, Dynamical Business & Science Society–DBSS International SAS, Bogotá 110311, Colombia; 5 Department of Psychiatry, The Panama Clinic, Pacific Center, Panama City 0831, Panama; dra.marilynmanzzo@gmail.com

**Keywords:** Alzheimer’s disease, *APOE*, Italy

## Abstract

**Objective:** This meta-analysis with a systematic review was undertaken to assess the association between *APOE* allelic genotypes and the risk of Alzheimer’s disease (AD) in the Italian population. **Methods:** The Web of Science, PubMed, and Scopus databases were searched until 15 November 2023. The odds ratio (OR) with a 95% confidence interval (CI) was calculated using fixed and random effect models, depending on the I^2^ statistic value. The systematic review and meta-analysis were conducted in agreement with the PRISMA guideline and registered with PROSPERO (CRD42023492580). **Results:** Our meta-analysis based on 15 studies revealed a higher risk of AD among Italian individuals carrying the *APOE* ε4 allele (OR = 3.60, 95% CI [2.90–4.47], *p* < 0.0001). The association of AD genotype *APOE* ε2ε4 (OR = 1.36, 95% CI [0.76–2.41], *p* = 0.29) was not statistically significant, while *APOE* ε3ε4 (OR = 3.43, 95% CI [2.95–3.99], *p* < 0.0001) has a high risk of AD development; the risk is more notably in the *APOE* ε4ε4 genotype (OR = 7.08, 95% CI [4.22–11.86], *p* < 0.0001). The *APOE* ε2 allele has a protective effect (*APOE* ε2 (OR = 0.47, 95% CI [0.29–0.74], *p* = 0.0013)), and similar results were achieved by *APOE* ε3 (OR  =  0.49, 95% CI [0.37–0.65], *p* < 0.0001). Subgroup analysis of three areas of Italy (southern, northern, and center) revealed that that *APOE ε*4 allele was a risk factor with a higher OR in northern Italy (OR 4.22; 95% CI [3.46–5.16], *p* < 0.0001) compared to southern and center Italy (OR 3.02; 95% CI [2.28–4.01], *p* < 0.0001 and OR 3.97; 95% CI [1.37–11.56], *p* < 0.0001, respectively). As well, *APOE ε4ε4* genotype carriers had a significantly higher OR in northern Italy (OR 9.69; 95% CI [4.94–18.99], *p* < 0.0001) compared to in southern and center Italy (OR 4.38; 95% CI [1.54–12.47], *p* < 0.0001 and OR 3.59; 95% CI [0.87–14.86], *p* < 0.0001, respectively). **Conclusions**: This systematic review with a meta-analysis of the Italian population on *APOE* alleles, genotyping, and AD incidence, highlights that individuals harboring *APOE* ε4 have a higher risk of developing AD compared to those with other alleles. It also supports the protective effect of the *APOE* ε2 allele against the progress of AD. The qualitative analysis on the complex genetic interactions influencing Alzheimer risk emphasizes the need for further research on genetic and environmental factors for effective prevention strategies.

## 1. Introduction

Alzheimer’s disease (AD) is a progressive neurodegenerative disorder and the most common cause of dementia, accounting for 60–80% of cases in elderly individuals [1]. Memory impairment, cognitive decline, and behavioral changes are most often associated with the disease [2,3], although less common clinical presentations are becoming more recognized. The core of AD neuropathology consists of extracellular beta-amyloid plaque deposits in discrete brain areas and intracellular neurofibrillary tangles (NFTs) composed of aggregated tau protein. Additional factors in the pathogenic framework of AD include the presence of neuroinflammatory conditions and vascular dysfunction [4]. Despite extensive research efforts, the etiology of AD remains poorly understood. Studies on familial forms of early-onset AD (EOAD) happening before 65 years of age have highlighted the crucial role of precise mutations in the amyloid precursor protein (*APP*), presenilin 1 (*PSEN1*), and presenilin 2 (*PSEN2*) genes; however, these rare familial forms of AD account for less than 1% of the cases [5]. More than 95% of AD cases are sporadic or late-onset AD (LOAD), where both environmental and genetic factors play a significant role in its etiology. The *APOE* gene has been identified as the most potent genetic risk factor in sporadic disease [6]. Located on chromosome 19q13.32, the *APOE* gene encodes a protein primarily involved in lipid metabolism and transport in the central nervous system [7,8]. Three allelic variants of the gene, namely ε2 (*APOE2*), ε3 (*APOE3*), and ε4 (*APOE4*), have a worldwide frequency of 8.4%, 77.9%, and 13.7%, respectively, and six genotypes (ε2ε2, ε2ε3, ε2ε4, ε3ε3, ε3ε4 and ε4ε4) [9] have been identified in humans. Meta-analyses have shown that the *APOE* ε4 allele increases the risk of the disease by three times in heterozygotes and by about 15 times in homozygotes [9]. In contrast, the *APOE* ε2 *allele* reduces AD risk by almost half, relative to the common *APOE ε3* allele [10,11]. Significantly, the *APOE ε4* both increases the risk of AD and lowers the age of disease onset in an allele number-dependent manner [12]. The precise molecular mechanism by which the *APOE* polymorphism determines the risk of AD remains unclear. Evidence advises that human *APOE* isoforms significantly impact AD pathogenesis through their differential effects on Aβ aggregation and clearance. However, *APOE* isoforms also affect multiple pathways that are not necessarily dependent on Aβ, with *APOE4* revealing either a gain in toxic function or a loss of physiological function [13]. Although the *APOE ε4* allele accounts for most of the genetic risk in sporadic AD [14], the contribution of other genes cannot be ruled out. Thanks to GWAS, dozens of genes robustly associated with AD have been recently discovered [15,16]. The heterogenic pathogenetic profile and the multifactorial nature of sporadic AD in which several susceptibility genes act in a complex interplay with environmental factors, might explain this difficulty [17]. Even if several questions are still open on the etiology of AD, the impact of genetic variants represents a key factor in the pathogenetic framework of the disease, as highlighted by several studies in the field. Herein, we conducted a meta-analysis to systematically assess the results of various research on the association among the *APOE* allele, genotypes, and AD risk for the first time in Italian population.

## 2. Materials and Methods

The systematic review and meta-analysis were conducted according to the Preferred Reporting Items for Systematic Reviews and Meta-Analyses (PRISMA) guidelines [18] and protocol registered at the PROSPERO International Prospective Registry (CRD42023492580). A machine learning approach was used using the MySLR platform [19]. MySRL is a semi-automated tool that implements the Latent Dirichlet Allocation LDA algorithm (https://myslr.unical.it, accessed on 1 November 2023). The LDA algorithm has been applied in some fields to extract topics and identify patterns within the literature [20,21].

### 2.1. Research Strategy

Two researchers (D.M.A-G and G.F.S) performed literature searches on the PubMed, Scopus, and Web of Science databases to identify publications in peer-reviewed journals up to 15 November 2023. The boolean operators “AND” and “OR” were used to combine the Medical Subject Heading (MeSH) terminology: (“apolipoprotein E” OR “*APO E*” OR “*APOE*”) AND (“italian” OR “italy”).

### 2.2. Paper Analysis and Data Extraction

The inclusion criteria for studies published up to 15 November 2023 were as follows: population: Italian population with AD; exposure: *APOE*; outcomes: present or absent allele and genotype; and types of study: case–control studies conducted on an Italian population. 

Articles were excluded if they were not case–control studies; were studies with an incomplete genotype or allele frequency; were not published in English; were review-based or non-original research; or involved non-human subjects. Disagreements were resolved by discussion or by third reviewer (M.C.C, M.M, and R.C). 

Two authors (D.M.A-G and G.F.S) extracted data, and one author (E.C.) checked them. The following data were extracted: authors and publication year, country, study design, participant characteristics, starting sample, diagnostic criteria, genotype and allele frequency, and result. 

### 2.3. Quality Assessment

The risk of bias of each study included was assessed using the NIH Study Quality Assessment Tool of Case–Control Studies (accessed 10 December 2023). Twelve questions were designed to help focus on the key concepts for evaluating each study’s internal validity. A traffic light plot and summary plot were created with the Robvis tool [22].

### 2.4. Statistical Analysis

Odds ratio (OR) with a 95% confidence interval (CI) and *p*-value were used to evaluate the relationship of *APOE* and AD in the following six genotypes and three allele frequencies. A Q test and the I^2^ were used to assess the heterogeneity of included studies [23]. The I^2^ test was used to determine the heterogeneity among the studies, and if I^2^ < 50%, then the studies were considered homogeneous, and the fit-equal model/fixed model (EE) was used to analyze the included data. When I^2^ ≥ 50% of the studies were considered heterogeneous, the random effect model (RE) was used to analyze the included data. A visual inspection of funnel plots and Egger’s test were used to assess the publication bias [24,25]. All statistical tests in this study were carried out using fixed and random effect designs implemented in the metafor R package, version 3.2.4 [26].

### 2.5. Results Presentation

The final step of the methodological approach is detailed in the ‘Results’ and ‘Discussion’ Sections. The latter is dedicated to defining and analyzing the results of the MySLR procedure and includes a meticulous review of the relevant literature to clearly describe and discuss the findings.

## 3. Results

**Figure 1:**
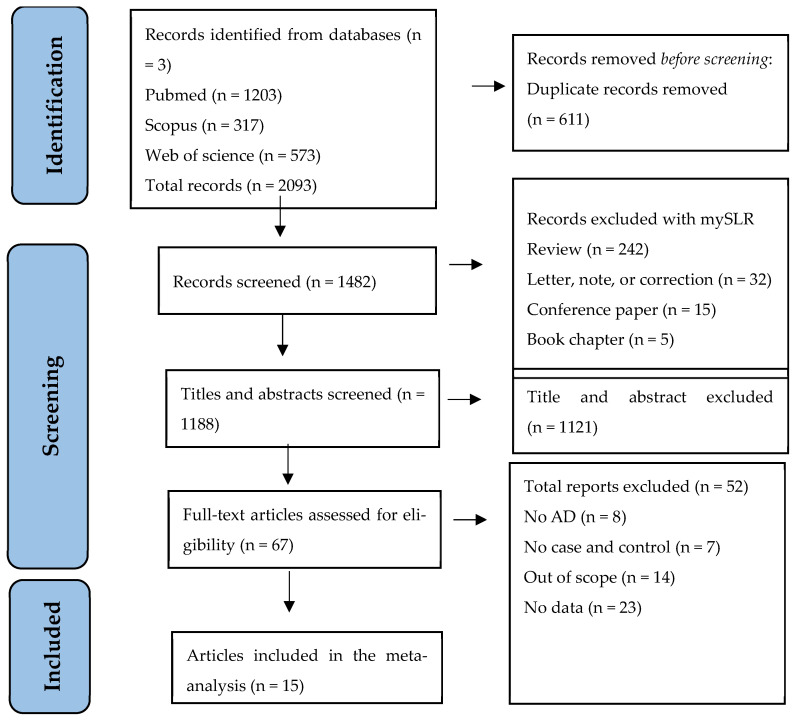
PRISMA Flow diagram for selecting eligible studies.

In total, 2093 records were identified from the initial literature search of the three databases (PubMed, Scopus, and Web of Science). After excluding duplicate records, 1482 items remained. MySLR removed 294 papers because they were review-based or non-original research. Of the 1188 studies remaining, 1121 were discarded based on their abstracts. After full-text reading and analysis, 52 records were excluded for failing to meet the inclusion/exclusion criteria. Therefore, a total number of 15 studies were considered eligible. The PRISMA flowchart in Figure 1 shows the selection of the studies for this systematic review.

### 3.1. Study Characteristics

The characteristics of the distribution of the selected studies are summarized in Table 1. Fifteen articles were selected, including 2846 cases and 2391 controls from three different areas of Italy: six studies from northern Italy [27,28,29,30,31,32], three studies from the center area [33,34,35], five studies from southern Italy [36,37,38,39,40,41], and only one study from a non-reported geographical area [42]. Blood was used for genomic DNA extraction in 13 articles [27,28,29,30,31,32,33,34,35,36,37,39,40], and two studies did not report the biological fluid that was used [38,42]. NINCDS-ADRDA was used as diagnostic criteria in 12 articles [27,28,29,30,32,33,35,36,37,38,39,40]. At the same time, three studies used DMS III/IV [35,37,38], and Nacmias et al. used a clinical/neurological examination, a laboratory test, and CT/MRI [31].

The global cognitive function was report through the Mini-Mental State Evaluation (MMSE) score by eight studies in both groups (case and control) [27,28,30,34,36,37,38] and solely in the control group of Lovati et al. and Bizzarro et al. [29,33]; Nacmias et al. did not report the MMSE score for the control group [31]. The distribution of the genotypes in the groups was consistent with the Hardy–Weinberg equilibrium (HWE). The distributions of alleles and genotypes in the individual studies are presented in Table 2.

### 3.2. Quality Assessment and Publication Bias

**Figure 2:**
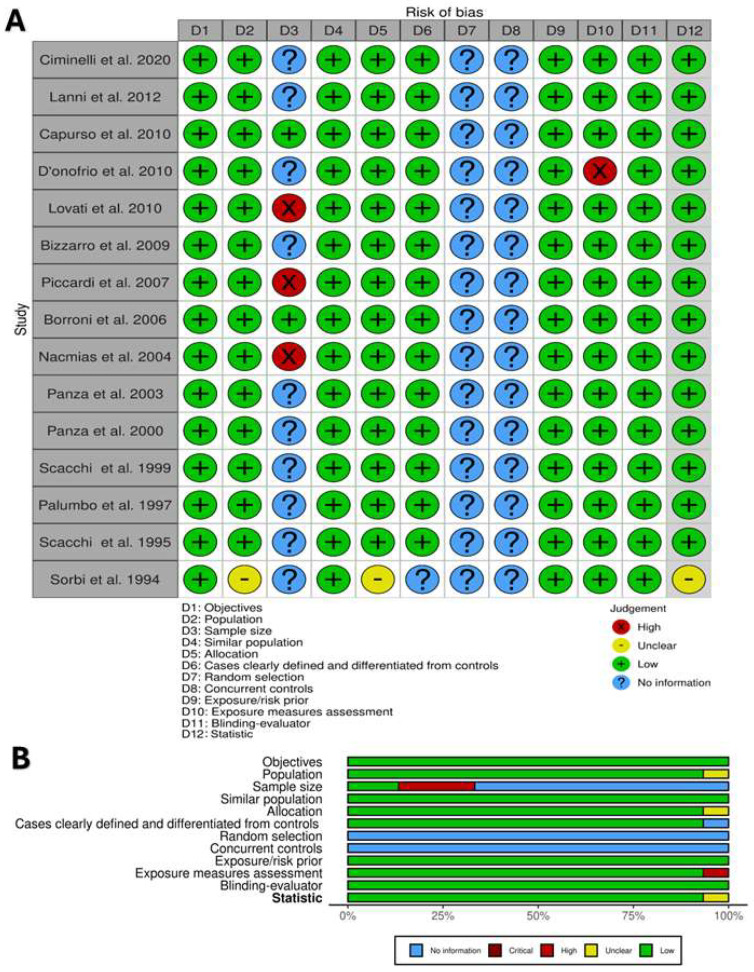
Risk of bias assessment for included cases and control studies. (**A**) Review of author’ judgment about each risk-of-bias item. (**B**) Presented ad percentage across all included studies. Created with Robvis tool.

The risk of bias assessment is summarized in Figure 2A,B. The total number of studies (100%) described objectives, similar populations, cases clearly defined and distinguished from controls, and the blinding of assessors, and they were judged to have a ‘fair’ and low risk of bias in these areas. No studies reported information about random selection and concurrent controls. Only two studies reported sample size justification [30,36]. All other domains were evaluated to have a low risk of bias. 

**Figure 3:**
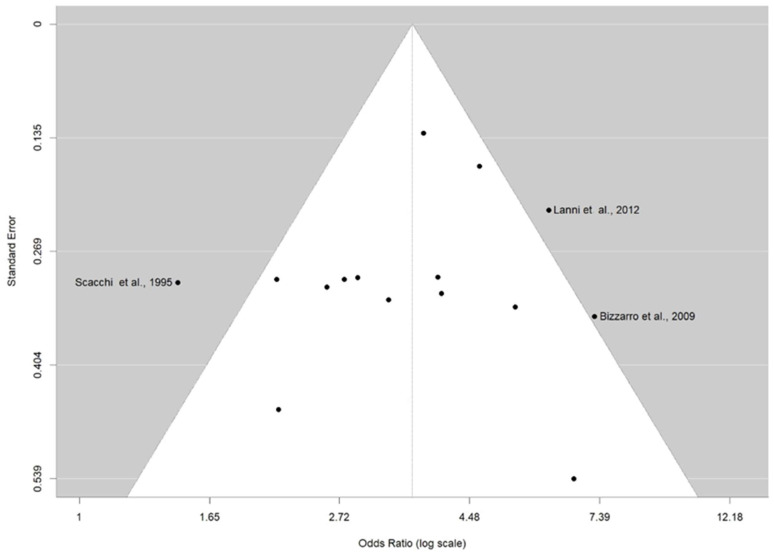
Funnel plot for publication bias on the association between ε4 allele carriers and AD risk. The *x*-axis displays the estimated effect size as OR (log scale), and the *y*-axis represents a measure of study precision as standard error. The horizontal line in the figure represents the overall estimated effects as the odds ratio (log scale). The two diagonal lines represent the 95% confidence limits of the effect estimate.

**Figure 4:**
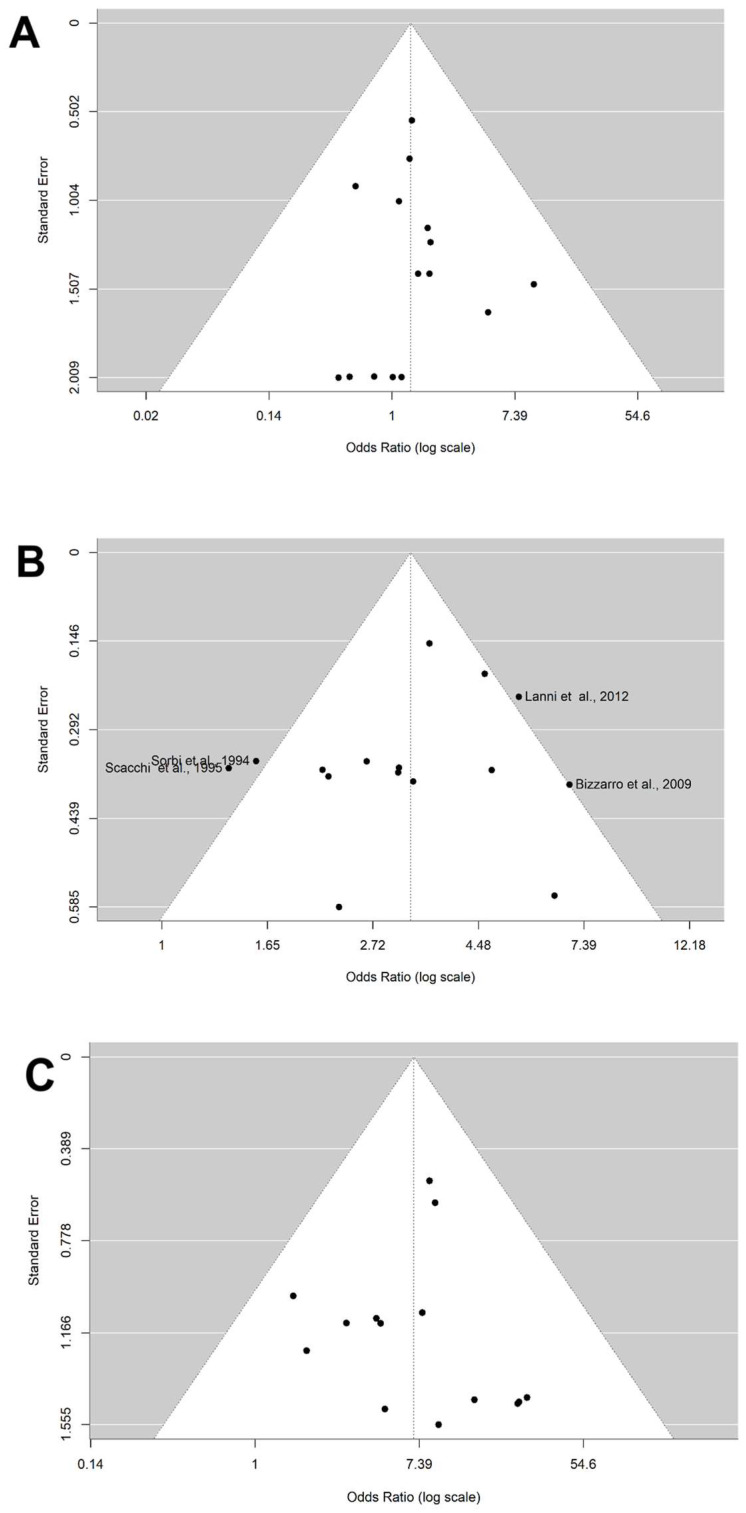
Funnel plot on the association between genotype carriers ε4 and AD risk. (**A**) Genotype ε2/ε4. (**B**) Genotype ε3/ε4. (**C**) Genotype ε4/ε4. The *x*-axis displays the estimated effect size as the OR (log scale), and the *y*-axis represents a measure of study precision as the standard error. The horizontal line in the figure represents the overall estimated effects as the odds ratio (log scale). The two diagonal lines represent the 95% confidence limits of the effect estimate.

The distribution of the ORs from individual studies in relation to their respective standard error in funnel plot is shown in Figure 3 and Figure 4A–C. A visual inspection of the funnel plots and Egger’s test for allele ε4 (*p* = 0.686) and genotypes ε2/ε4 (0.916), ε3/ε4 (*p* = 0.461), and ε4/ε4 (*p* = 0.895) revealed no evidence of asymmetry. Thus, there was no possibility of publication bias risk in the meta-analysis.

### 3.3. Meta-Analysis of APOE Alleles and AD

The I^2^  >  50% and Q statistics were significant; thus, heterogeneity between studies was significant (Table 3). 

The random effect model (RE) was applied for calculating the pooled OR. The results of each allele of *APOE* in this meta-analysis are listed in Table 4. The results show a significant association among *APOE* ε2 (OR = 0.47, 95% CI [0.29–0.74], *p* = 0.0013), *APOE* ε3 alleles, and AD (OR  =  0.49, 95% CI [0.37–0.65], *p* < 0.0001), highlighting the lower risk of AD for the ε2 and ε3 alleles.

**Figure 5:**
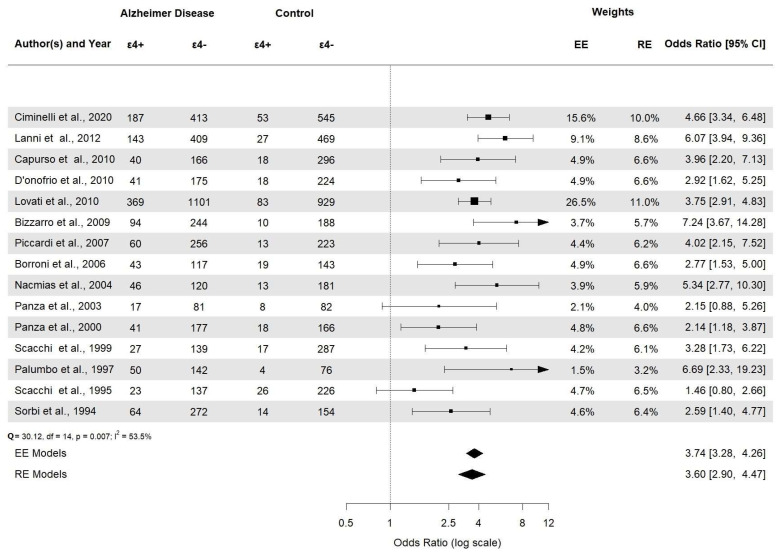
Forest plot of the association between *APOE* ε4 alleles and AD risk in Italian population.

All forest plots are included in the Supplementary Material. Overall, this meta-analysis displays that the frequency of the *APOE* ε4 allele is higher in AD than in the healthy controls and established a statistically significant association among risk factor *APOE* ε4 allele carriers and AD in the Italian population (OR = 3.60, 95% CI [2.90–4.47], *p* < 0.0001) (Figure 5).

### 3.4. Meta-Analysis of APOE Genotypes and AD

With the exception of the *APOE* ε2ε2 genotype, *APOE ε2ε2*, and *APOE* ε4ε4 homozygotes, all I^2^  >  50% and Q statistics were significant (Table 5).

Therefore, the corresponding fit-equal model (EE) and random model (RE) were used for calculating the pooled OR. The results of each allele of *APOE* in this meta-analysis are listed in Table 6.

**Figure 6:**
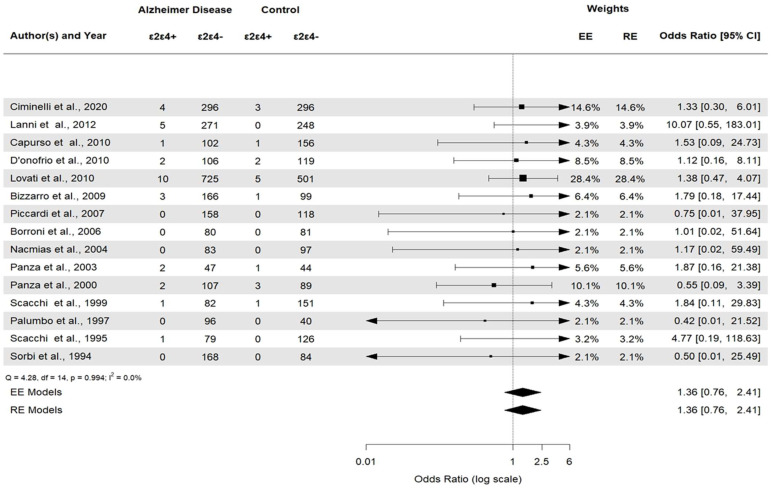
Forest plot of the association between *APOE* ε2/ε4 genotypes and AD versus control.

**Figure 7:**
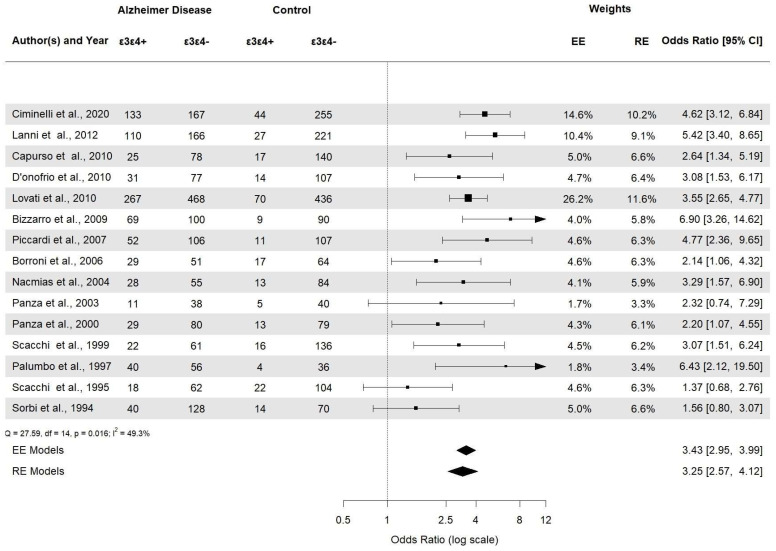
Forest plot of the association between *APOE* ε3/ε4 genotypes and AD risk.

**Figure 8:**
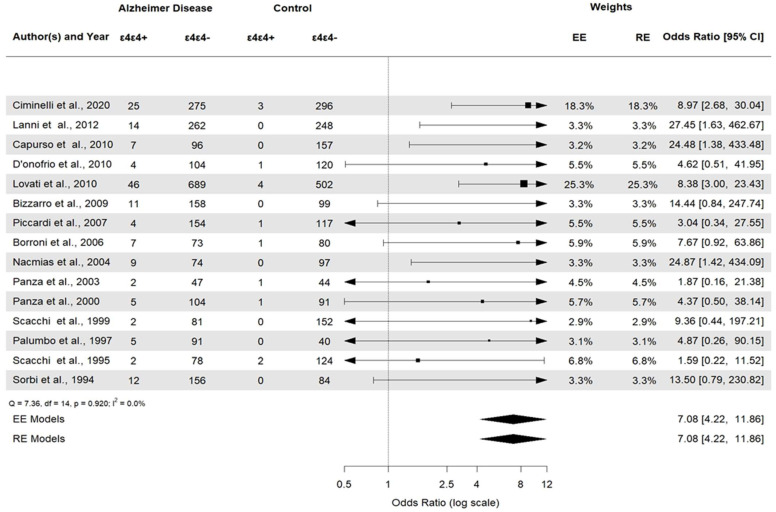
Forest plot of the association between *APOE* ε4/ε4 genotypes and AD.

*APOE* ε2ε3 was associated with AD (*APOE* ε2ε3: OR 0.38, 95% CI [0.22–0.66], *p* = 0.0006). *APOE* ε2/ε2 and *APOE* ε2/ε4 (Figure 6) were not statistically significant (*p* = 0.23 and *p* = 0.29, respectively). Then, the *APOE* ε3ε3 and *APOE* ε3ε4 (Figure 7) genotypes were also significantly associated with AD (*APOE* ε3ε3: OR 0.47, 95% CI [0.33–0.67], *p* < 0.0001). Genotype investigation showed a higher frequency of *APOE* ε3/ε4 genotypes in AD patients (OR 3.43, 95% CI [2.95–3.99], *p* < 0.0001) and of *APOE* ε4/ε4 in AD patients compared to controls (OR  =  7.08, 95% CI [4.22–11.86]), *p* < 0.0001), indicating a strong association with AD patients (Figure 8). The OR of *APOE* ε4ε4 homozygotes was the largest, and that of *APOE* ε2ε3 was the smallest. All forest plots are included in the Supplementary Material.

### 3.5. Subgroup Analysis of APOE ε4 Allele and APOE ε4ε4 Genotype Based on Location

**Figure 9:**
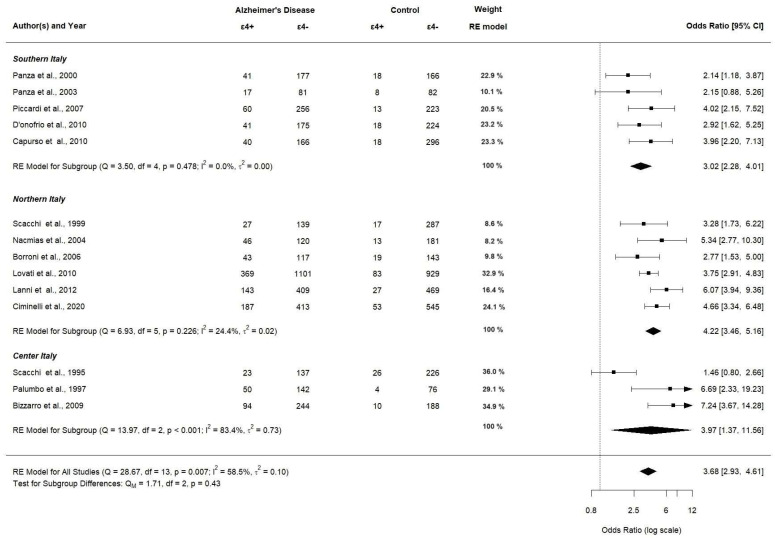
Subgroup analysis of *APOE ε4* allele (assessed in 14 studies).

When stratified by location (southern, northern, and center), we used only 14 articles due to Sorbi et al. not reporting an allocation study [42]. *APOE ε4* allele carriers significantly had a higher OR in northern Italy (OR 4.22; 95% CI [3.46–5.16], *p* < 0.0001) compared to southern and center Italy (OR 3.02; 95% CI [2.28–4.01], *p* < 0.0001 and OR 3.97; 95% CI [1.37–11.56], *p* < 0.0001, respectively). A forest plot of the subgroup analyses by location is displayed in Figure 9.

**Figure 10:**
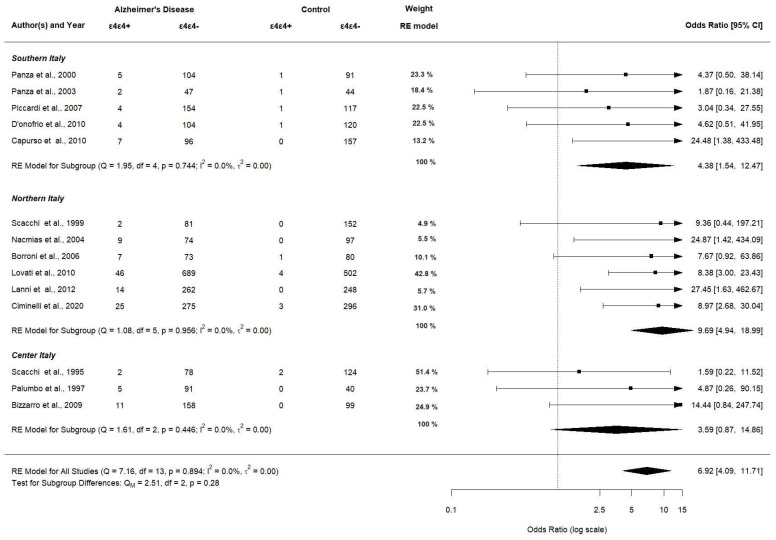
Subgroup analysis of *APOE ε4ε4* allele (assessed in 14 studies).

Figure 10 shows the corresponding results for *APOE ε4ε4* genotype carriers significantly having a higher OR in northern Italy (OR 9.69; 95% CI [4.94–18.99], *p* < 0.0001) compared to southern and center Italy (OR 4.38; 95% CI [1.54–12.47], *p* < 0.0001 and OR 3.59; 95% CI [0.87–14.86], *p* < 0.0001, respectively). 

## 4. Discussion

AD is a complex neurodegenerative disorder in which genetic variants are implicated in its pathogenesis. While mutations in *APP, PSEN1*, and *PSEN2* genes lead to the occurrence of EOAD, variants in lots of other genes have been correlated with an augmented risk of developing a LOAD form of the disease. Among these, *APOE* ε4 was first described as a major risk factor for AD in *1993* [43]. A study published by Farmer et al. shows that the effects of *APOE* ε4 induce lipid droplet formation and cholesterol accumulation in astrocytes, which are specialized cells that, among their various functions, supply nutrients to neurons and support the structure of the brain [44,45]. While *APOE ε2* has been shown to confer protection against AD-related pathology [13]. According to a previous meta-analysis, the prevalence of the *APOE* ε4 genotype differs between AD patients by geographical region and sample size [46], with northern Europe having the highest estimates and Asia and southern Europe having the lowest. In Europe, *APOE4* frequency increases with latitude from the highest frequencies in Norway (31.0%) to the lowest frequencies in Italy (Sardinia: 5.2%) [46]. Therefore, several meta-analyses have been performed in certain ethnic groups to examine the relation between *APOE* ε4 and AD. Liu et al. reported that the *APOE* ε4 allele and *APOE ε4ε4* genotype are related with 3.93-fold and 11.76-fold increased risks of developing AD, respectively, in a Chinese population [47]. Also, Agarwal et al. conducted a meta-analysis on an Indian population and showed that the risk of developing AD increased 5.90-fold and 4.81-fold for individuals carrying the *APOE* ε4 allele or with the *APOE* ε4ε4 genotype [48]. Similarly, Abyadeh and co-workers suggested an association between the *APOE* ε4 allele and ε4ε4 genotype and the risk of developing AD in an Iranian population (4.81- and 7.47-fold, respectively), while pointing out a protective role of the *APOE* ε3 allele [49]. An association between *APOE ε4* and increased AD and related dementia risk was recently reported in global Hispanic populations, with the strength of this association varying across Hispanic subgroups [50].

The present meta-analysis included 15 cases and control studies, comprising 2391 patients in the experimental group and 2583 in the control group. Herein, we conducted a meta-analysis of studies exploring the connection between *APOE* haplotypes and AD in the Italian population. Overall, our results show that the risk of developing AD in ε4 allele carriers was 3.60-fold higher than *APOE ε4* noncarriers. We also found a statistically significant association among *APOE* ε2, *APOE ε3,* and AD with a low positive rate ((OR = 0.47; 95% CI [0.29–0.74], *p* = 0.0013; OR = 0.49; 95% CI [0.37–0.65], *p* < 0.0001, respectively)) for both alleles, thus underscoring their lower risk of developing AD. The results also reveal that the risk of developing AD in individuals with the *APOE ε4ε4* genotype was 7.08-fold higher than *APOE* ε4ε4 genotype non-carriers. Notably, *APOE ε4* homozygosity has been recently proposed as another form of genetically determined AD, owing to near-full penetrance, the predictability of symptom onset, and a probable sequence of biomarker and clinical changes [51]. This evidence could lead to a conceptual reshaping of the genetic architecture of AD, which is usually categorized into the sporadic and autosomal dominant forms, with important consequences for public health and genetic counseling. Our results also reveal that *APOE ε3* and *APOE ε4* heterozygotes show an intermediate OR value (OR = 3.43; 95%CI [2.95–3.99], *p* < 0.0001) between *APOE ε3* (OR = 0.47; 95% CI [0.33–0.67], *p* < 0.0001) and *APOE ε4* homozygotes (OR = 7.08; 95% CI [4.22–11.86], *p* < 0.0001), suggesting a dose-dependent association among the number of *APOE* ε4 alleles and the risk of AD in the Italian population. No significant association was found between the *APOE* ε2ε4 allele and AD risk. Interestingly, although *APOE ε2* has been shown to confer protection against AD [52], Insel et al. reported that carrying an *APOE ε2* in the presence of *APOE ε4* may confer some defense against Aβ accumulation compared to *APOE ε3* [53,54,55]. However, other studies found that the OR of *APOE* ε2ε4 individuals is more comparable to that of *APOE* ε4 carriers than *APOE* ε2 carriers [52], suggesting that the increased risk associated with *APOE* ε4 is more dominant than the protection offered by *APOE* ε2. Moreover, the relationship between the *APOE* genotype and AD is not only determined by genetic factors but is also modulated by complex gene–environment interactions such as diet, physical activity, cognitive stimulation, and cardiovascular health, which may modify the risk conferred by the *APOE* genotype; for example, Panza et al. reported a significant reduction in serum APOE levels from *APOE ε2* to *ε4* carriers. In addition, serum HDL levels among age groups significantly decreased in ε4 carriers only [39]. Furthermore, Borroni et al. reported that subjects with hypercholesterolemia and heterozygous for the *APOE ε4* allele had more than a three-fold risk to develop AD compared with the control group [30], highlighting the importance of adopting preventive strategies. The latter can be recognized in life style and nutrition; in particular, a mediterranean diet rich in antioxidants and omega-3 is associated with fewer signs of AD in the brains of older adults [56]. Additionally, supplements with antioxidants and other micronutrients can boost cognitive function [57,58]. Finally, in the subgroup analysis by region, the association of the *APOE ɛ4ɛ4* genotype with AD was striking. In the three areas of Italy (southern, northern, and center), the ɛ4 allele was associated with more or less the same risk of AD compared with controls, but the estimates of the association of the ɛ4ɛ4 genotypes by subgroups show an approximately double risk in northern Italy compared with southern and center Italy. In this context, the meta-analysis across the global population of AD patients recruited in southern Europe/Mediterranean appeared to have a significantly lower ε4 carrier status compared to in northern Europe. This difference may be due to the different sample sizes of the studies, to the specific geographical characteristics, or to different gene–environment interactions. According to a national study, southern Italian regions shown high adherence to the Mediterranean diet compared to northern regions [59]. However, in recent years, there has been a progressive decline in adherence to the Mediterranean diet in the South of Italy [60]. Although several environmental factors and lifestyle can also play a part in the modification of AD risk, no single environmental/lifestyle risk factor has been shown to be strongly associated with AD yet [61]. Recently, a Polygenic Risk Score based on 21 SNPs for AD contributed a modifier of risk of the *APOE ε4* homozygotes, unlike other factors that contributed more to causality [62,63]. In our systematic review, Bizzarro et al. pointed out that rs449647 A/T and rs405509 T/T genotypes were negative to AD, and rs449647 A/A and rs405509 G/G genotypes were positive. This means that promoter genotypes and *APOE* haplotypes might have a complex function in AD-associated genetic risk factors [33]. Interestingly, the modifying effect of the rs405509 genotype explained much of the ethnic variability in the AD/*APOE ε4* association [64]. In addition, Lanni et al. reported a synergy between the *COMT* Val158Met GG genotype and *APOE ε4*, which increased to about a 2–3-fold risk for AD, markedly in males [28]. Finally, Capurso et al. founded no interaction or synergy between *GSTO1* SNP and *APOE* variants [36]. These findings demonstrate the potential impact of rare *APOE* variants on AD risk. 

This is the first systematic review and meta-analysis to specifically investigate the effects of *APOE* alleles and genotypes associated with AD in the Italian population. The quality of the clinical trials was evaluated, and most of the included studies had a good quality. Also, no evidence of publication bias was evidenced. Specific eligibility criteria and controlled queries were applied to the literature search, and we are confident that the search strategy retrieved all relevant studies. However, there are some limitations in this meta-analysis. Firstly is heterogeneity across studies, due to differences in aims. Secondly, eligible studies could not be investigated because of inadequate evidence regarding the potential effects of other parameters such as gender, age, cognitive status, and interactions with environmental factors such as diet or physical activity. Thirdly, the retrospective nature of case–control studies limits causal inference and requires caution in interpreting the results. Finally, few studies have reported total and fractionated cholesterol values, which would provide important information regarding the potential causality of associations between total cholesterol, *APOE* genotype, and AD risk.

## 5. Conclusions

In conclusion, our meta-analysis with a systematic review suggests that *APOE ε4* carriers are associated with AD in the Italian population, and supports the protective effect of the *APOE ε2* allele against developing AD. In addition, the estimates of the association of the ɛ4ɛ4 genotypes by subgroup analysis show an approximately double risk of AD in the northern Italy population compared with in the south and center.

## Figures and Tables

**Table 1:** Qualitative and quantitative synthesis and characteristics of the studies.

Study	Location	Design Study	Cases/Controls	Starting Sample	Diagnostic Criteria	MMSE Test Score	Ref.
Ciminelli B.M. et al. (2020)	Northern	Case–control	300/299	Blood	NINCDS-ADRDA	AD = 17.03 ± 6.12 C = 28.79 ± 1.06	[27]
Lanni C. et al. (2012)	Northern	Case–control	276/248	Blood	NINCDS-ADRDA	AD = 15 ± 8 C = 28 ± 2	[28]
Capurso C. et al. (2010)	Southern	Case–control	103/157	Blood	NINCDS-ADRDA	AD = 16 (14–18) C = 27 (26–29)	[36]
D’Onofrio G. et al. (2010)	Southern	Case–control	108/121	Blood	NINCDS-ADRDA DSM-IV	AD = 13.81 ± 5.23 C = 28.07 ± 1.70	[37]
Lovati et al. (2010)	Northern	Case–control	735/506	Blood	NINCDS-ADRDA	AD = NR C => 28–30	[29]
Bizzarro et al. (2009)	Center	Case–control	169/99	Blood	NINCDS-ADRDA	AD = NR C = >28/30	[33]
Piccardi et al. (2007)	Southern	Case–control	158/118	NR	NINCDS-ADRDA DSM-IV	AD = 20.1 ± 3.71 C = 26	[38]
Borroni et al. (2006)	Northern	Case–control	80/81	Blood	NINCDS-ADRDA	AD = 19.3 ± 6 C = >27	[30]
Nacmias et al. (2004)	Northern	Case–control	83/97	Blood	Clinical/neurological examination, laboratory test, and CT/MRI	AD = 21.2 ± 4.4 C = NR	[31]
Panza et al. (2003)	Southern	Case–control	49/45	Blood	NINCDS-ADRDA	NR	[39]
Panza et al. (2000)	Southern	Case–control	109/92	Blood	NINCDS-ADRDA	NR	[40]
Panza et al. (1999)	Southern	Case–control	79/192	Blood	NINCDS-ADRDA	NR	[41]
Scacchi et al. (1999)	Northern	Case–control	83/152	Blood	NINCDS-ADRDA	NR	[32]
Palumbo B. et al. (1997)	Center	Case–control	96/40	Blood	NINCDS-ADRDA	AD = 15 ± 5 C = ≥26	[34]
Scacchi R. et al. (1995)	Center	Case–control	80/126	Blood	NINCDS-ADRDA DMS-III	NR	[35]
Sorbi S. et al. (1994)	NR	Case–control	168/84	NR	NR	NR	[42]

AD: Alzheimer’s disease; C: control; NR: not reported.

**Table 2:** *APOE* allele and genotype frequencies in AD case and control groups.

Study	Groups	n	Allele Frequencies			Genotype Frequencies						Ref.
			ε2	ε3	ε4	ε2ε2	ε2ε3	ε2ε4	ε3ε3	ε3ε4	ε4ε4	
Ciminelli et al. (2020)	Cases	300	10	403	187	0	6	4	132	133	25	[27]
	Controls	299	33	512	53	0	30	3	219	44	3	
Lanni et al. (2012)	Cases	276	22	387	143	0	17	5	130	110	14	[28]
	Controls	248	20	449	27	0	20	0	201	27	0	
Capurso et al. (2010)	Cases	103	5	161	40	0	4	1	66	25	7	[36]
	Controls	157	22	274	18	1	19	1	119	17	0	
D’Onofrio et al. (2010)	Cases	108	8	167	41	0	6	2	65	31	4	[37]
	Controls	121	18	206	18	0	16	2	88	14	1	
Lovati et al. (2010)	Cases	735	41	1060	369	1	29	10	382	267	46	[29]
	Controls	506	65	864	83	3	54	5	370	70	4	
Bizzarro et al. (2009)	Cases	169	13	231	94	0	10	3	76	69	11	[33]
	Controls	99	17	171	10	0	16	1	73	9	0	
Piccardi et al. (2007)	Cases	158	2	254	60	0	2	0	100	52	4	[38]
	Controls	118	5	218	13	0	5	0	101	11	1	
Borroni et al. (2006)	Cases	80	3	114	43	0	3	0	41	29	7	[30]
	Controls	81	5	138	19	0	5	0	58	17	1	
Nacmias et al. (2004)	Cases	83	7	113	46	0	7	0	39	28	9	[31]
	Controls	97	7	174	13	0	7	0	77	13	0	
Panza et al. (2003)	Cases	49	4	77	17	0	2	2	32	11	2	[39]
	Controls	45	23	59	8	1	20	1	17	5	1	
Panza et al. (2000)	Cases	109	5	172	41	0	3	2	70	29	5	[40]
	Controls	92	28	138	18	1	23	3	51	13	1	
Scacchi et al. (1999)	Cases	83	8	131	27	0	7	1	51	22	2	[32]
	Controls	152	13	274	17	0	12	1	123	16	0	
Palumbo et al. (1997)	Cases	96	0	142	50	0	0	0	51	40	5	[34]
	Controls	40	11	65	4	0	11	0	25	4	0	
Scacchi et al. (1995)	Cases	80	5	132	23	0	4	1	55	18	2	[35]
	Controls	126	15	211	26	0	15	0	87	22	2	
Sorbi et al. (1994)	Cases	168	40	232	64	0	40	0	76	40	12	[42]
	Controls	84	8	146	14	0	8	0	62	14	0	

**Table 3:** Test of heterogeneity in meta-analysis of allele *APOE* and risk of AD.

Outcome	Test of Heterogeneity		
Allele	Q	I^2^ (%)	*p*
ε2	51.49	72.8	<0.001
ε3	70.69	80.2	<0.001
ε4	30.12	53.5	0.007

**Table 4:** Pooled OR results in meta-analysis of *APOE* allele and risk of AD.

Outcome	Test of Association		
Allele	OR	95% CI	*p*
ε2	0.47	0.29–0.74	0.0013
ε3	0.49	0.37–0.65	<0.0001
ε4	3.60	2.90–4.47	<0.0001

**Table 5:** Test of heterogeneity in meta-analysis of genotype *APOE* and risk of AD.

Outcome	Test of Heterogeneity		
Genotypes	Q	I^2^ (%)	*p*
ε2 ε2	2.07	0.0	1.000
ε2 ε3	56.23	75.1	<0.001
ε2 ε4	4.28	0.0	0.99
ε3 ε3	76.20	81.6	<0.001
ε3 ε4	27.59	49.3	0.01
ε4 ε4	7.36	0.0	0.92

**Table 6:** Pooled OR results in meta-analysis of *APOE* genotype and risk of AD.

Outcome	Test of Association		
Genotypes	OR	95% CI	*p*
ε2 ε2	0.57	0.23–1.43	0.23
ε2 ε3	0.38	0.22–0.66	0.0006
ε2 ε4	1.36	0.76–2.41	0.29
ε3 ε3	0.47	0.33–0.67	<0.0001
ε3 ε4	3.43	2.95–3.99	<0.0001
ε4 ε4	7.08	4.22–11.86	<0.0001

## Data Availability

All data generated/analyzed throughout this research are included in this article.

## Supplementary Materials

The following supporting information can be downloaded at https://www.mdpi.com/article/10.3390/brainsci14090908/s1: Table S1: PRISMA 2020 checklist. Figure S1: Forest plot of the association between *APOE* ε2 allele [65]; Figure S2: Forest plot of the association between *APOE* ε3 allele; Figure S3: Forest plot of the association between *APOE* ε2ε2 genotype; Figure S4: Forest plot of the association between *APOE* ε2ε3 genotype; Figure S5: Forest plot of the association between *APOE* ε3ε3 genotype; Figure S6: Forest plot of the association between *APOE* ε3ε4 genotype.
